# Sublethal Effects of Chlorantraniliprole on the Mobility Patterns of *Sitophilus* spp.: Implications for Pest Management

**DOI:** 10.3390/insects15060451

**Published:** 2024-06-13

**Authors:** Nickolas G. Kavallieratos, Maria C. Boukouvala, Nikoleta Eleftheriadou, Constantin S. Filintas, Demeter Lorentha S. Gidari, Vasiliki Panagiota C. Kyrpislidi

**Affiliations:** Laboratory of Agricultural Zoology and Entomology, Faculty of Crop Science, Agricultural University of Athens, 75 Iera Odos Str., 11855 Athens, Greece; mbouk@aua.gr (M.C.B.); nikolelef@aua.gr (N.E.); p1172219@aua.gr (C.S.F.); dlgidari@aua.gr (D.L.S.G.); vassokyrp@gmail.com (V.P.C.K.)

**Keywords:** stored-product pests, anthranilic diamide, locomotion, walking

## Abstract

**Simple Summary:**

This study investigates the sublethal effects of chlorantraniliprole, an insecticide known for its low toxicity to mammals and selectivity towards non-target organisms, on two significant stored-product pests, *Sitophilus oryzae* (L.) and *Sitophilus zeamais* Motschulsky (Coleoptera: Curculionidae). Through contact toxicity assays, differences in susceptibility between the two species are observed, with *S. zeamais* showing higher sensitivity. Subsequent analysis reveals altered mobility behavior in chlorantraniliprole-exposed groups compared with controls, particularly in *S. oryzae*, which displays reduced number of food approaches and altered locomotion patterns. In contrast, *S. zeamais* exhibits increased walking time and decreased immobility periods under sublethal concentrations. These findings underscore the importance of considering sublethal effects in understanding the overall impact of chlorantraniliprole on stored-product pest populations. Further exploration into the long-term consequences of sublethal exposure is recommended to enhance pest management strategies.

**Abstract:**

Chlorantraniliprole, an anthranilic diamide insecticide, has emerged as a promising solution for controlling agricultural pests because of its low mammalian toxicity and selectivity towards non-target organisms. This study investigated the sublethal effects of chlorantraniliprole on the mobility behavior of two significant stored-product pests, *Sitophilus oryzae* (L.) and *Sitophilus zeamais* Motschulsky (Coleoptera: Curculionidae). Contact toxicity assays revealed varying susceptibility levels between the two species, with *S. zeamais* showing higher sensitivity. Subsequent analysis of mobility behavior, both in the presence and absence of food, indicated significant differences between chlorantraniliprole-exposed and control groups. While *S. oryzae* exhibited altered locomotion patterns and a decreased number of food approaches at sublethal concentrations, *S. zeamais* displayed increased walking time and reduced immobility periods. These findings highlight the importance of considering sublethal effects in understanding the overall impact of chlorantraniliprole on stored-product pests. Further research into the long-term consequences of sublethal exposure is warranted to inform more effective pest management strategies in storage.

## 1. Introduction

*Sitophilus oryzae* (L.) and *Sitophilus zeamais* Motschulsky (Coleoptera: Curculionidae), commonly known as the rice weevil and the maize weevil, respectively, are two major primary pests in the global agriculture industry [1,2]. These pests typically thrive in tropical, subtropical, and temperate climate zones. They have been documented to infest various commodities, including grains, pulses, processed cereal products, nuts, pasta, and fruits [3,4]. Their similar morphologies and life cycles are often associated with the marked preferred commodity they develop in, with *S. oryzae* primarily infesting wheat and *S. zeamais* predominantly affecting maize [4,5]. Both insects infiltrate grains to deposit their eggs, resulting in a decrease in the quality and weight of the commodities. This process is often accompanied by a foul odor, rendering the product unsuitable for commercial use, while it frequently attracts and contributes to the proliferation of secondary pests [6,7].

For decades, numerous synthetic insecticides have been employed in the control of stored-product pests and the deterioration they induce [8]. Organophosphorus insecticides have been widely used across the globe to manage agricultural pests. For example, chlorpyrifos stands as one of the frequently utilized organophosphorus compounds, earning classification as a moderately hazardous substance concerning human and animal health and, consequently, the environment [9,10]. In addition to organophosphates, several pyrethroid compounds have been employed in grain storage facilities to safeguard against pests in various countries [11]. Pyrethroids are extensively employed in agricultural and public health because of their relatively low mammalian toxicity, great insecticidal efficacy at low concentrations, and quick knockdown effects [12,13,14]. The impacts of several pyrethroids, notably, deltamethrin and cypermethrin, have been investigated concerning stored-product pests, primarily through grain treatment [11,15,16,17].

Besides inducing mortality in storage pests, synthetic pesticides also induce sublethal effects that can influence the biology and behavioral characteristics of insects [18,19]. Some of the sublethal effects include aggressive behavior and a negative impact on courtship, lifespan, fecundity, fertility, locomotion, and morphology [20,21,22]. Insects have developed a range of behavioral reactions to insecticidal substances, leading to their reduced efficacy [23]. For instance, exposure to dry insecticide residues impacted the movement ability of *Halyomorpha halys* Stål (Hemiptera: Pentatomidae), a major agricultural pest in the United States [24]. Specifically, it was suggested that organophosphate insecticides had a neutral effect on the horizontal mobility of *H. halys*, whereas pyrethroids drastically inhibited the horizontal mobility of adult insects. Furthermore, Morrison et al. [25] demonstrated that treating commodities such as wheat, rice, and corn with label doses of synthetic compounds reduced movement by 50–88% in adults exposed to each insecticide formulation compared with untreated controls.

Chlorantraniliprole (3-bromo-*N*-[4-chloro-2-methyl-6-[(methylamino) carbonyl] phenyl]-1-(3-chl-oro-2-pyridinyl)-1*H*-pyrazole-5-carboxamide) is an anthranilic diamide insecticide, exerting its effects through the modulation of insect ryanodine receptors. This modulation results in the depletion of intracellular calcium stores, leading to disrupted muscle regulation, paralysis, and, ultimately, the demise of the insect [26]. Chlorantraniliprole exhibits low mammalian toxicity and selectivity towards non-target organisms (i.e., pollinators, parasitoids, and predators) [27]. To date, chlorantraniliprole has been assessed and proven effective in inducing mortality against a broad spectrum of insect pests of agricultural significance across various orders, including beetles, moths or butterflies, true bugs, flies, termites, and thrips [28,29,30,31,32]. The insecticidal effectiveness and suppression of progeny by chlorantraniliprole have been previously evidenced for *S. oryzae*, exhibiting effectiveness in immediate and delayed mortality [33,34], along with its efficacy and persistence against *S. zeamais* when combined with lambda-cyhalothrin [35]. The sublethal effects of this promising compound have been studied for several coleopteran pests [36,37,38,39,40,41,42]. Nevertheless, the sublethal effects of chlorantraniliprole are yet to be determined for *Sitophilus* spp., among other significant stored-product pests. Hence, the current study aims to ascertain, for the first time, the impact of sublethal concentrations of chlorantraniliprole on the mobility behavior of two important stored-product pests, *S. oryzae* and *S. zeamais*, to comprehensively explore all facets of the efficacy of this compound in combatting significant stored-product pests.

## 2. Materials and Methods

### 2.1. Insecticidal Formulations

The formulation of chlorantraniliprole, Altacor WG (wettable granules), with 350 g/kg active ingredient (a.i.) was used for the experiments. The formulation was purchased from Greece by Dupont (Athens, Greece).

### 2.2. Sitophilus oryzae and Sitophilus zeamais Rearing

The insect species used in the experiments originated from cultures maintained at the Laboratory of Agricultural Zoology and Entomology, Agricultural University of Athens, Greece, since 2003, for approximately 240 generations. The colonies were kept in total darkness at 30 °C and 65% relative humidity (RH) [43,44]. The rearing medium comprised uncontaminated hard wheat kernels [45] and maize kernels [46] devoid of infestation or impurities and had not undergone any prior treatment for *S. oryzae* and *S. zeamais,* respectively.

### 2.3. Bioassays on Contact Toxicity

To evaluate the contact toxicity of chlorantraniliprole against the adults of *S. oryzae* and *S. zeamais*, six series of chlorantraniliprole dilutions (i.e., 0.01, 0.025, 0.05, 0.1, 0.25, and 0.5 a.i./cm^2^) were prepared in distilled water to assess the LC_10_, LC_30,_ and LC_50_ concentrations. Filter papers (Whatman No. 1, Sigma-Aldrich Chemie GmbH, Taufkirchen, Germany) were placed separately on the bottom of Petri dishes, which measured 8 cm in diameter and 1.5 cm in height. Using a micropipette, each filter paper was impregnated with 1 mL of each chlorantraniliprole solution corresponding to the above dilutions and left to dry for 120 min at 30 °C. A total of 5 replications (5 replications × 6 concentrations = 30 dishes per species) were performed for each concentration. Consequently, 20 adults of *S. zeamais* or *S. oryzae*, less than two weeks old, were placed in each dish. Following that, the dishes were placed in incubators set at 30 °C and 65% RH and continuous darkness. The count of deceased adults of *S. zeamais* and *S. oryzae* in the dishes was conducted 72 and 96 h post-exposure, respectively. Control dishes were also prepared by impregnating filter papers with distilled water only, as described above (5 replications, 5 dishes per species).

### 2.4. Sublethal Effects on Mobility

The adults of *S. oryzae* and *S. zeamais* used in the following experiments were exposed to LC_10_ and LC_30_ of chlorantraniliprole for 24 h, following the procedure described in Section 2.3.

#### 2.4.1. Mobility in the Absence of Food

A plastic, 80 mm in diameter Petri dish (50.27 cm^2^ in surface) was used for the experiment. Filter paper (Whatman No. 1) was placed on the bottom of each dish. Following this, a single adult of either *S. oryzae* or *S. zeamais* from the control group, LC_10_-exposed group, or LC_30_-exposed group was introduced into the center of the dish (arena). Each adult was allowed a three-minute acclimatization period within the arena. Subsequently, the following traits were visually recorded: (1) “walking (s)”—the duration of locomotion, (2) “stops (s)”—the duration of immobility, (3) “climb up (s)”—the duration of attempts to ascend the arena walls, (4) “upturned (s)”—the duration of time spent on the insect’s back, (5) “short stops (n)”—the number of interruptions in movement followed by resumption, (6) “climb up (n)”—the number of attempts to ascend the arena walls, (7) “upturned (n)”—the number of instances the beetle was found on its back, (8) “flying attempts (n)”—the number of flight attempts into the arena, and (9) “flying”—the duration of flight within the arena [47]. These observations were conducted for 15 min. Each adult exposed to chlorantraniliprole LC_10_, LC_30_, or the control underwent thirty replicates. Separate arenas were utilized for each replication, species, and exposure. The experiments were conducted between 8 a.m. and 8 p.m. at a temperature of 30 °C [47].

#### 2.4.2. Mobility in the Presence of Food

In the present experiment, the locomotion behavior of *S. oryzae* and *S. zeamais* adults exposed to chlorantraniliprole LC_10_, LC_30_, or the control was observed in the presence of food. For this purpose, 0.3 g of wheat kernels for *S. oryzae* and 0.3 g of maize kernels for *S. zeamais* were positioned at the center of the arena. Prior to the bioassays, insects were deprived of food for an entire day. Subsequently, an adult of either *S. oryzae* or *S. zeamais*, exposed to chlorantraniliprole LC_10_, LC_30_, or the control, was released near the edge of the arena (the dish’s walls), and the time taken to reach the food source was recorded. Additionally, the duration spent on the food patch and the number of subsequent visits to the food were noted. Thirty replicates were conducted for each of the *S. oryzae* and *S. zeamais* LC_10_-exposed, LC_30_-exposed, or control groups, with behavioral observations conducted visually over a period of 15 min. The experiments were carried out between 8 a.m. and 8 p.m. at a temperature of 30 °C.

### 2.5. Statistical Analysis

Using probit analysis, sublethal concentrations corresponding to the LC_10_, LC_30,_ and LC_50_ values of chlorantraniliprole in adults of *S. oryzae* and *S. zeamais* were determined independently with a 95% confidence interval (CI) [48,49]. R statistical software (version 2.15.1) was used to determine the sublethal concentrations of chlorantraniliprole [50]. Data on the impact of chlorantraniliprole on the walking behavior of *S. oryzae* and *S. zeamais* adults were transformed to log(x + 1) format to achieve standard means and normal variances [47,51]. Two-way ANOVA was conducted to assess the impact of chlorantraniliprole on the mobility of both weevil species [52]. The Tukey HSD test was employed to separate means at a significance level of 0.05 [53] using the statistical package JMP 16.2 [54].

## 3. Results

### 3.1. Contact Toxicity on S. oryzae and S. zeamais

According to concentration–response assays, *S. zeamais* adults were more susceptible to chlorantraniliprole compared with *S. oryzae* adults (Table 1). For *S. zeamais* adults, the LC_10_, LC_30_, and LC_50_ values of chlorantraniliprole were 0.000319, 0.00249, and 0.0103 mg a.i./cm^2^, respectively, while for *S. oryzae* adults, the LC_10_, LC_30_, and LC_50_ values were higher, i.e., 0.000328, 0.00411, and 0.0236 mg a.i./cm^2^, respectively.

### 3.2. Mobility in the Absence of Food

The main effect species was significant for all tested traits, while concentration was significant for upturned (n), flying (n), and flying (s). The interaction species × concentration was not significant for walking (s), stops (n), and stops (s) (Table 2). For all traits tested, no significant differences were noticed compared to controls, apart from the number of climb-ups for LC_30_-exposed adults and the duration of climbing for both the LC_10_- and LC_30_-exposed *S. oryzae* adults (Table 3). For the LC_30_-exposed adults, the number of times they attempted to climb the arena’s walls was significantly lower compared with the adults of the control (5.4 and 10.2 for LC_30_ and control, respectively). The adults treated with LC_10_ and LC_30_ spent significantly less time attempting to climb the walls (158.2 s and 130.2 s for LC_10_ and LC_30_, respectively) than the control adults (294.1 s). Furthermore, LC_10_ and LC_30_ caused the exposed individuals to lie on their dorsal side for longer than the control weevils (20.0 s, 26.1 s, and 13.9 s, respectively), while flight attempts were observed only in the control group.

Regarding the adults of *S. zeamais* exposed to LC_30_ of chlorantraniliprole or control, significant differences were noticed in all the traits tested. The time the weevils spent walking was significantly increased for the LC_10_-exposed and LC_30_-exposed adults (569.5 s and 535.9 s, respectively) compared with the control adults (413.1 s). The number of times that the insects stopped walking as well as the duration the insects were motionless was significantly reduced for the LC_10_-exposed (2.2 and 81.6 s, respectively) and LC_30_-exposed adults (1.2 s and 38.8 s, respectively) compared with the control (5.3 s and 292.7 s, respectively). The duration of climbing attempts in the arena’s walls showed a significant increase for the LC_30_-exposed adults (309.3 s) compared with the LC_10_-exposed (225.2 s) and control adults (202.2 s). Only the adults from the control and LC_10_ groups performed flying attempts (Table 4).

### 3.3. Mobility in the Presence of Food

The main effect species was not significant for all three traits tested, while concentration was significant for food approach. The interaction species × concentration was significant for all three traits (Table 5). The time spent between the arena and the food source differed significantly for the LC_10_-exposed *S. oryzae* adults (677.2 s) and control (868.3 s), while the differences were not significant between the control and the LC_30_-exposed adults (825.7 s) (Table 6). The time that *S. oryzae* adults spent in food was significantly higher in LC_10_ (225.2 s) than in the controls (74.6 s), whereas LC_30_ (33.5 s) did not demonstrate significant differences with the control. Regarding the number of times the weevil approached the food source, a significantly lower number of visits were observed in LC_30_ (0.5) compared with the control (1.5) and LC_10_ (1.5). Concerning *S. zeamais,* no significant differences were observed among adults treated with LC_10_, LC_30_, or the control for any of the traits tested. However, the duration of the time spent between the arena and the food source was higher in LC_10_ (846.1 s), followed by the control (783.9 s) and LC_30_ (754.5 s), while less time was spent in the food source by the LC_10_-treated weevils (53.0 s) than the controls (102.5 s) and LC_30_-treated weevils (121.8 s) (Table 7).

## 4. Discussion

Species of *Sitophilus* pose a major threat to the food industry worldwide because of their expansion capabilities through global commerce and the immense post-harvest crop losses they cause [55,56]. A rising global problem lies in insecticide-resistant strains of *Sitophilus* spp., on both organophosphates and pyrethroids, in regions of Egypt, Brazil, Australia, and South Korea [8,57,58,59]. Furthermore, their life cycles as primary pests require larvae to develop inside the kernel, rendering their control efforts difficult depending on the substrate [60]. Especially for *S. oryzae*, Boukouvala et al. [61] demonstrated that etofenprox used on several grain commodities provides different mortality rates for adults of this weevil. For example, the highest mortality was recorded on treated barley (95.0%), while for oats, maize, wheat, and whole rice, the treatment resulted in decreased mortality levels, varying from 56.7 to 80.6%, 21 days post-exposure.

Aside from toxicity-focused mortality bioassays, the sublethal effects of insecticidal formulations on behavioral traits, like mobility implications, may play an essential role in insect pest control [62]. Several insecticides, like chlorantraniliprole, interfere with the locomotor capacities of insects, leading to their demise [63]. According to the results of the present study regarding walking behavior, when both lethal concentrations were tested with the presence of food, the *S oryzae* group in LC_10_ spent significantly less time between the arena and the food source, as well as on the food source only, compared with the controls. In contrast, the results for *S. zeamais* demonstrate that the LC_10_-exposed beetles spent more time between the arena and the food source and less time in the food source than the control group; nonetheless, the differences were not significant. This emphasizes that the exposure of the beetles to chloranthraniliprole did not influence the duration spent on the food source. However, evaluating whether this behavior originated from foraging or eating is outside the purview of this work and merits further research. In addition, varying walking values were observed, and no flying attempts were recorded for the exposed beetles, compared to the control. Specifically, LC_30_ led to fewer climbing attempts and reduced climbing duration in *S. oryzae* compared with the controls. LC_10_ and LC_30_ caused *S. oryzae* adults to spend more time on their dorsal side. *Sitophilus zeamais* demonstrated increased walking duration with LC_10_ and LC_30_, alongside fewer stops and longer stop periods compared with the controls. LC_30_ significantly affected climbing and upturned behavior in *S. zeamais*. Flying behavior was significantly affected, eliminating it in both sublethal concentrations for *S. zeamais* individuals. It is therefore concluded that there is no specific trend in the response, as it seems that once these beetles were exposed to the active compound, they became more active in terms of walking, climbing, and decreasing stop durations. These findings suggest that these two sister species [64] react variably in contact with the same insecticide at sublethal concentrations. They also shed light on important negative effects of chlorantraniliprole, especially in lower concentrations, that can affect the motor activity and flying capacity of *S. oryzae* and eventually their population ecology. Reduced climbing and the lack of flying attempts can obstruct the overall colony development of *S. oryzae* and *S. zeamais*, and potentially other stored-product pests, through reduced foraging and mating, as well as prevent new infestations nearby [25]. Contrarily to the control group, *S. zeamais* individuals exposed to this insecticide demonstrated increased mobility or variable flying activity and spent longer durations upturned, indicating an immediate irritation effect.

After a thorough literature review, it is deduced that there has been significant effort in understanding the flight/walking behavior after insecticide exposure in a plethora of insect pests, including species of the genus *Sitophilus*. For example, Morrison et al. [65] highlighted that the ability of *H. halys* adults to move horizontally, climb vertically, and fly was reduced by an average of 20–60% when exposed to insecticides compared with a control group. In particular, methomyl, thiamethoxam, and thiamethoxam + lambda-cyhalothrin maintained over 65% climbing capacity, while thiamethoxam and bifenthrin preserved over 50% walking capacity. However, after exposure to insecticides, a considerable number of *H. halys* adults maintained notable mobility and flight capability, with flight being particularly noticeable immediately following exposure. In a recent study by Silva Barros et al. [66], male moths of *Chloridea virescens* (Fabricius) (Lepidoptera: Noctuidae) exposed to chlorantraniliprole demonstrated shorter flight distances in comparison with a control group. Furthermore, the flight speed of males was significantly reduced after chlorantraniliprole treatment. Another related study on the lethal and sublethal effects of chlorantraniliprole against *Anticarsia gemmatalis* Hübner (Lepidoptera: Erebidae), a major pest of soybean crops, demonstrated that LD_50_ proved effective in reducing the overall walking ability of the tested individuals. It notably reduced the velocity as well as the total distance covered by the larvae [67]. Previously, Guedes et al. [68] conducted a study on the flight take-off and walking behavior of both resistant and susceptible strains of *S. zeamais*, demonstrating that behavioral responses to deltamethrin differed among strains regardless of concentration, with resistance to stimuli unrelated to physiological resistance. Males showed varying flight take-off rates, while females exhibited consistent mobility. The authors concluded that behavioral resistance did not always correlate with physiological resistance. According to de Araújo et al. [69], the number of take-offs observed in the tested groups of *S. zeamais* remained consistent when exposed to essential oils, similar to the control group. The authors noted varying levels of flight activity among different resistant strains when exposed to insecticides, highlighting the response variations among different strains of the same species [69]. Although our study documented lower or zero flight activity of both *Sitophilus* species tested compared with the control, this important finding needs further investigation by testing more strains of *S. oryzae* and *S. zeamais* with the same and additional insecticides. Given that both species coexist in storage [70], the prohibition of their flight activity with the application of sublethal concentrations of a single insecticide minimizes the probability of their dispersal, especially *S. zeamais* since it is a much stronger flyer than *S. oryzae* [71]. It has been reported that *S. zeamais* exhibits a heightened flight activity and natural dispersal ability within storage units, suggesting the potential for migration between storage areas and fields, and vice-versa [71,72,73].

Here, contact toxicity tests demonstrated that for the adults of *S. zeamais,* significantly lower concentrations of chlorantraniliprole were required to achieve mortality for LC_10_, LC_30_, and LC_50_. In a former study, Vásquez-Castro et al. [74] suggested that the increased tolerance to fenitrothion mixed with esfenvalerate exhibited in *S oryzae* comparatively to *S. zeamais* is a direct outcome of the behavioral differences in each species. Specifically, *S. zeamais* has a greater flight capability, promoting cross-infestations, which would lead to increased gene flow in certain populations, reducing tolerance levels for a specific insecticide [74]. On the other hand, *S. oryzae* is mostly abundant in warehouses and is frequently exposed to pesticides, leading to selective pressures and favoring tolerance evolution [75]. The differences in the susceptibility of *S. zeamais* compared with *S. oryzae* are in line with the above observations, nevertheless, irrespective of tolerance.

## 5. Conclusions

To our knowledge, this is the first time that the sublethal effects of chloranthraniliprole on *S. oryzae* and *S. zeamais* have been studied. The results of this investigation provide useful data regarding the susceptibility of *S. zeamais* in comparison to *S. oryzae* to low concentrations of this compound. Our research revealed significant differences in susceptibility between these congeneric species, with *S. zeamais* exhibiting increased motility and altered behavioral patterns compared with *S. oryzae* when exposed to chlorantraniliprole. This study underscores the importance of considering sublethal effects, such as affected mobility and behavior, alongside mortality rates when assessing the efficacy of insecticides on pest control. Understanding these nuances is crucial for developing effective integrated pest management strategies that minimize economic losses and mitigate the development of insecticide resistance. Furthermore, our findings highlight the need for continued research into the sublethal effects of insecticides on stored-product insect pests, particularly in species with global economic significance.

## Figures and Tables

**Table 1:** Contact toxicity of chlorantraniliprole on *S. oryzae* and *S. zeamais* adults.

Insect Species	Concentration	LC_10_ (95% CI)	LC_30_ (95% CI)	LC_50_ (95% CI)	*χ*^2^, df, *p*
*Sitophilus oryzae*	mg a.i./cm^2^	0.000328 (0.0000440–0.00106)	0.00411 (0.00136–0.00800	0.0236 (0.0136–0.0349)	30.7, 28, 0.331
*Sitophilus zeamais*	mg a.i./cm^2^	0.000319 (0.0000583–0.000907)	0.00249 (0.000867–0.00484)	0.0103 (0.00547–0.0159)	21.3, 28, 0.811

LC = lethal concentration that kills 10%, 30%, and 50% of the exposed beetles. 95% CI = lower and upper limits of the 95% confidence interval.

**Table 2:** ANOVA parameters for main effects and associated interactions for the mobility traits of *S. oryzae and S. zeamais* adults exposed to LC_10_ and LC_30_ of chlorantraniliprole (total df = 119).

Mobility Traits	Species			Concentration			Species × Concentration		
	df	*F*	*p*	df	*F*	*p*	df	*F*	*p*
Walking (s ^1^)	1	7.9	0.01 *	1	0.1	0.9	1	0.3	0.61
Stops (n ^2^)	1	16.8	<0.01 *	1	2.8	0.10	1	0.7	0.42
Stops (s ^1^)	1	24.2	<0.01 *	1	2.7	0.10	1	0.5	0.49
Climbing (n ^2^)	1	34.8	<0.01 *	1	0.2	0.66	1	6.0	0.02 *
Climbing (s ^1^)	1	22.8	<0.01 *	1	0.1	0.91	1	3.8	0.05 *
Upturned (n ^2^)	1	25.9	<0.01 *	1	5.8	0.02 *	1	7.7	<0.01 *
Upturned (s ^1^)	1	13.7	<0.01 *	1	3.2	0.08	1	4.9	0.03 *
Flying (n ^2^)	1	5.3	0.02 *	1	5.3	0.02 *	1	5.3	0.02 *
Flying (s ^1^)	1	5.7	0.02 *	1	5.7	0.02 *	1	5.7	0.02 *

^1^ Seconds. ^2^ Number. * Significant.

**Table 3:** Mobility traits of *S. oryzae* exposed to LC_10_ and LC_30_ of chlorantraniliprole. Values are means (±standard errors). Within each column, different letters indicate significant differences (Tukey HSD test, *p* < 0.05). The absence of letters indicates no significant differences among values (Total df = 2.89).

Treatment	Walking (s ^1^)	Stops (n ^2^)	Stops (s ^1^)	Climbing (n ^2^)	Climbing (s ^1^)	Upturned (n ^2^)	Upturned (s ^1^)	Flying (n^2^)	Flying (s ^1^)
Control	450.0 ± 33.8	3.3 ± 0.5	153.6 ± 38.7	10.2 ± 0.9 a	294.1 ± 28.0 a	2.7 ± 0.6	13.9 ± 4.1	0.6 ± 0.53	15.5 ± 14.0
LC_10_	483.1 ± 42.4	3.0 ± 0.3	260.1 ± 56.5	8.4 ± 1.3 ab	158.2 ± 22.7 b	3.3 ± 0.8	20.0 ± 5.8	0.0 ± 0.0	0.0 ± 0.0
LC_30_	446.9 ± 48.0	3.0 ± 0.5	324.5 ± 63.3	5.4 ± 0.9 b	130.2 ± 23.0 b	3.1 ± 0.7	26.1 ± 7.0	0.0 ± 0.0	0.0 ± 0.0
*F*	0.24	0.19	0.80	6.10	7.29	0.013	0.04	1.49	1.96
*p*	0.784	0.824	0.449	0.033 *	0.012 *	0.987	0.957	0.232	0.146

^1^ Seconds. ^2^ Number. * Significant.

**Table 4:** Mobility traits of *S. zeamais* exposed to LC_10_ and LC_30_ of chlorantraniliprole. Values are means (±standard errors). Within each column, different letters indicate significant differences (Tukey HSD test, *p* < 0.05) (Total df = 2.89).

Treatment	Walking (s ^1^)	Stops (n ^2^)	Stops (s ^1^)	Climbing (n ^2^)	Climbing (s ^1^)	Upturned (n ^2^)	Upturned (s ^1^)	Flying (n ^2^)	Flying (s ^1^)
Control	413.1 ± 30.3 b	5.3 ± 0.39 a	292.7 ± 41.0 a	9.3 ± 0.9 b	202.2 ± 19.7 b	3.8 ± 0.6 b	19.2 ± 4.6 b	1.9 ± 0.8 a	23.8 ± 14.9 a
LC_10_	569.5 ± 26.1 a	2.2 ± 0.5 b	81.6 ± 20.6 b	11.9 ± 1.1 ab	225.2 ± 22.3 b	5.4 ± 1.0 b	28.0 ± 5.6 ab	0.3 ± 0.1 ab	2.0 ± 0.9 ab
LC_30_	535.9 ± 34.6 a	1.2 ± 0.3 b	38.8 ± 10.8 b	14.7 ± 0.9 a	309.3 ± 27.8 a	10.2 ± 1.2 a	45.1 ± 5.9 a	0.0 ± 0.0 b	0.0 ± 0.0 b
*F*	5.80	28.50	21.68	6.07	3.69	12.21	8.25	5.64	4.8
*p*	0.004 *	<0.01 *	<0.01 *	0.034 *	0.029 *	<0.01 *	0.05 *	0.01 *	0.01 *

^1^ Seconds. ^2^ Number. * Significant.

**Table 5:** ANOVA parameters for main effects and associated interactions for the walking traits of *S. oryzae and S. zeamais* adults exposed to LC_10_ and LC_30_ of chlorantraniliprole in the presence of a food source (total df = 119).

Walking Traits	Species			Concentration			Species × Concentration		
	df	*F*	*p*	df	*F*	*p*	df	*F*	*p*
Time between arena and food	1	1.1	0.30	1	2.3	0.14	1	12.8	<0.01 *
Time spent in food	1	1.4	0.25	1	2.6	0.11	1	11.4	<0.01 *
Food approach	1	2.1	0.15	1	3.9	0.05 *	1	6.1	0.01 *

* Significant.

**Table 6:** Effect on the walking parameters of *S. oryzae* with food in the center of the arena of the exposure to LC_10_ and LC_30_ of chlorantraniliprole. Values are means (±standard errors). Within each column, different letters indicate significant differences (Tukey HSD test, *p* < 0.05) (Total df = 2.89).

Treatment	Time between Arena and Food (s ^1^)	Time Spent in Food (s ^1^)	Food Approach (n ^2^)
Control	825.7 ± 23.0 a	74.6 ± 23.4 b	1.5 ± 0.3 a
LC_10_	677.2 ± 52.2 b	225.2 ± 52.0 a	1.5 ± 0.3 a
LC_30_	868.3 ± 14.6 a	33.5 ± 15.8 b	0.5 ± 0.1 b
*F*	7.55	7.17	5.92
*p*	0.009 *	0.013 *	0.039 *

^1^ Seconds. ^2^ Number. * Significant.

**Table 7:** Effect on the walking parameters of *S. zeamais* with food in the center of the arena of the exposure to LC_10_ and LC_30_ of chlorantraniliprole. Values are means (±standard errors). Within each column, different letters indicate significant differences (Tukey HSD test, *p* < 0.05). The absence of letters indicates no significant differences among values (Total df = 2.89).

Treatment	Time between Arena and Food (s ^1^)	Time Spent in Food (s ^1^)	Food Approach (n ^2^)
Control	783.9 ± 38.7	102.5 ± 33.2	0.2 ± 0.04
LC_10_	846.1 ± 24.9	53.0 ± 24.4	0.2 ± 0.04
LC_30_	754.5 ± 42.6	121.8 ± 38.0	0.2 ± 0.04
*F*	1.61	0.80	0.09
*p*	0.206	0.451	0.913

^1^ Seconds. ^2^ Number.

## Data Availability

The data are available within this article.

## References

[1] Hill D.S. (2003). Pests of Stored Foodstuffs and Their Control.

[2] Rees D.P. (2004). Insects of Stored Products.

[3] Hagstrum D. (2016). Atlas of Stored-Product Insects and Mites.

[4] Kumar D., Kalita P. (2017). Reducing postharvest losses during storage of grain crops to strengthen food security in developing countries. Foods.

[5] Devi S.R., Thomas A., Rebijith K.B., Ramamurthy V.V. (2017). Biology, morphology and molecular characterization of *Sitophilus oryzae* and *S. zeamais* (Coleoptera: Curculionidae). J. Stored Prod. Res..

[6] Trematerra P., Sciarreta A., Tamasi E. (2000). Behavioural responses of *Oryzaephilus surinamensis*, *Tribolium castaneum* and *Tribolium confusum* to naturally and artificially damaged durum wheat kernels. Entomol. Exp. Appl..

[7] Upadhyay R.K., Ahmad S. (2011). Management strategies for control of stored grain insect pests in farmer stores and public ware houses. WJAS.

[8] Kim B., Song J.E., Park J.S., Park Y., Shin E.M., Yang J. (2019). Insecticidal effects of fumigants (EF, MB, and PH3) towards phosphine-susceptible and-resistant *Sitophilus oryzae* (Coleoptera: Curculionidae). Insects.

[9] John E.M., Shaike J.M. (2015). Chlorpyrifos: Pollution and remediation. Environ. Chem. Lett..

[10] Wołejko E., Łozowicka B., Jabłońska-Trypuć A., Pietruszyńska M., Wydro U. (2022). Chlorpyrifos occurrence and toxicological risk assessment: A review. Int. J. Environ. Res. Public Health.

[11] Kavallieratos N.G., Athanassiou C.G., Arthur F.H. (2015). Efficacy of deltamethrin against stored-product beetles at short exposure intervals or on a partially treated rice mass. J. Econ. Entomol..

[12] World Health Organization (2005). Safety of Pyrethroids for Public Health Use.

[13] Palmquist K., Salatas J., Fairbrother A., Perveen F.K. (2012). Pyrethroid insecticides: Use, environmental fate, and ecotoxicology. Insecticides-Advances in Integrated Pest Management.

[14] Arthur F.H. (2018). Residual efficacy of deltamethrin as assessed by rapidity of knockdown of *Tribolium castaneum* on a treated surface: Temperature and seasonal effects in field and laboratory settings. J. Stored Prod. Res..

[15] Vayias B.J., Kavallieratos N.G., Athanassiou C.G., Tatsi G. Insecticidal action of the combined use of spinosad and deltamethrin against three stored-product pests in two stored hard-wheat varieties. Proceedings of the 10th International Working Conference on Stored Product Protection.

[16] Arthur F.H. (2019). Residual efficacy of a deltamethrin emulsifiable concentrate formulation against *Rhyzopertha dominica* (F.) and *Sitotroga cerealella* (Oliver) after partial treatment of brown rice. Insects.

[17] Arthur F.H., Domingue M.J., Scheff D.S., Myers S.W. (2019). Bioassays and methodologies for insecticide tests with larvae of *Trogoderma granarium* (Everts), the khapra beetle. Insects.

[18] Guedes R.N.C., Guedes N.M.P., Rosi-Denadai C.A. (2011). Sub-lethal effects of insecticides on stored-product insects: Current knowledge and future needs. Stewart Postharvest Rev..

[19] Zinhoum R. (2020). Sublethal Effects of malathion on biology and population growth of khapra beetle, *Trogoderma granarium* Everts (Coleoptera: Dermestidae). Egypt. Acad. J. Biol. Sci. A Entomol..

[20] Campbell B., Baldwin R., Koehler P. (2017). Locomotion inhibition of *Cimex lectularius* L. following topical, sublethal dose application of the chitin synthesis inhibitor lufenuron. Insects.

[21] Boukouvala M.C., Kavallieratos N.G., Žikić V., Stanković S.S., Ilić Milošević M., Skourti A., Lazarević M. (2023). Sub-lethal effects of pirimiphos-methyl are expressed to different levels in wings of three stored-product coleopterans: A geometric morphometrics investigation. Insects.

[22] Benelli G., Ceccarelli C., Zeni V., Rizzo R., Verde G.L., Sinacori M., Boukouvala M.C., Kavallieratos N.G., Ubaldi M., Tomassoni D. (2022). Lethal and behavioural effects of a green insecticide against an invasive polyphagous fruit fly pest and its safety to mammals. Chemosphere.

[23] Zalucki M.P., Furlong M.J. (2017). Behavior as a mechanism of insecticide resistance: Evaluation of the evidence. Curr. Opin. Insect Sci..

[24] Lee D.H., Wright S.E., Leskey T.C. (2013). Impact of insecticide residue exposure on the invasive pest, *Halyomorpha halys* (Hemiptera: Pentatomidae): Analysis of adult mobility. J. Econ. Entomol..

[25] Morrison W.R., Arthur F.H., Bruce A. (2021). Characterizing and predicting sublethal shifts in mobility by multiple stored product insects over time to an old and novel contact insecticide in three key stored commodities. Pest Manag. Sci..

[26] Cordova D., Benner E.A., Sacher M.D., Rauh J.J., Sopa J.S., Lahm G.P., Selby T.P., Stevenson T.M., Flexner L., Gutteridge S. (2006). Anthranilic diamides: A new class of insecticides with a novel mode of action, ryanodine receptor activation. Pestic. Biochem. Phys..

[27] Bhuvaneswari K., Mani M., Suganthi A., Manivannan A., Mani M. (2022). Novel insecticides and their application in the management of horticultural crop pests. Trends in Horticultural Entomology.

[28] Meesters C., Van Kerckvoorde V., Beliën T., Bylemans D., Herman L., Clymans R., Jacquemyn H., Lievens B. (2024). Efficacy of pesticides against *Nesidiocoris tenuis* Reuter (Hemiptera: Miridae), an emerging threat in the cultivation of tomato in Northwest Europe. Crop Prot..

[29] Hackmeyer E.J., Washburn T.J., Delaplane K.S., Bartlett L.J. (2023). Successful application of anthranilic diamides in preventing small hive beetle (Coleoptera: Nitidulidae) infestation in honey bee (Hymenoptera: Apidae) colonies. J. Insect Sci..

[30] Behera R.K., Muralimohan K. (2024). Seed treatment with diamides provides protection against early and mid-stage larvae of the fall armyworm, *Spodoptera frugiperda* (Lepidoptera: Noctuidae), in maize. J. Asia Pac. Entomol..

[31] Yadav S.P.S., Pokhrel S., Poudel A., Devkota S., Katel S., Bhattarai N., Gautam P. (2024). Evaluation of different insecticides against *Liriomyza sativae* (Diptera: Agromyzidae) on cucumber plants. J. Agric. Food Res..

[32] Akbar M.S., Sajjad F., Afzal M., Luqman M., Riaz M.A., Majeed M.Z. (2021). Field evaluation of promising botanical extracts, plant essential oils and differential chemistry insecticides against subterranean termites *Odontotermes obesus* (Isoptera: Termitidae). SJA.

[33] Boukouvala M.C., Kavallieratos N.G. (2021). Evaluation of two formulations of chlorantraniliprole as maize protectants for the management of *Prostephanus truncatus* (Horn) (Coleoptera: Bostrychidae). Insects.

[34] Kavallieratos N.G., Boukouvala M.C., Nika E.P., Eleftheriadou N., Avtzis D.N. (2021). Immediate and delayed mortality of four stored-product pests on concrete surfaces treated with chlorantraniliprole. Insects.

[35] Magano D.A., Carvalho I.R., Doberstein A.P., Louro M.V., Bubans V., Drebes L., Guedes J.V.C., Launtenchleger F., Ferreira L.L., Boller W. (2021). Efficiency and persistence of insecticides with different action mechanisms applied on wheat stored pest *Sitophilus zeamais*. Aust. J. Crop Sci..

[36] Nawaz M., Cai W., Jing Z., Zhou X., Mabubu J.I., Hua H. (2017). Toxicity and sublethal effects of chlorantraniliprole on the development and fecundity of a non-specific predator, the multicolored Asian lady beetle, *Harmonia axyridis* (Pallas). Chemosphere.

[37] Plata-Rueda A., Martínez L.C., Costa N.C.R., Zanuncio J.C., de Sena Fernandes M.E., Serrão J.E., Guedes R.N.C., Fernandes F.L. (2019). Chlorantraniliprole–mediated effects on survival, walking abilities, and respiration in the coffee berry borer, *Hypothenemus hampei*. Ecotoxicol. Environ. Saf..

[38] Jiang J., Wang Y., Mu W., Zhang Z. (2020). Sublethal effects of anthranilic diamide insecticides on the demographic fitness and consumption rates of the *Coccinella septempunctata* (Coleoptera: Coccinellidae) fed on *Aphis craccivora*. Environ. Sci. Pollut. Res..

[39] Khan M.M., Hafeez M., Elgizawy K., Wang H., Zhao J., Cai W., Ma W., Hua H. (2021). Sublethal effects of chlorantraniliprole on *Paederus fuscipes* (Staphylinidae: Coleoptera), a general predator in paddle field. Environ. Pollut..

[40] Xie W., Deng X., Tao W., Zhang Z., Zhang H., Li Q., Jiang C. (2024). Sublethal effects of chlorantraniliprole on immunity in *Spodoptera frugiperda* (Smith) (Lepidoptera: Noctuidae): Promote encapsulation by upregulating a heat shock protein 70 family gene *SfHSP68.1*. Pestic. Biochem. Phys..

[41] Ren H., Zhang H., Tan Y., Ni R., Shan Y., Li F., Dai G., Li F., Li Y., Pang B. (2024). Sublethal effects of chlorantraniliprole on biological characteristics, detoxifying enzyme activity and gene expression profile in the *Allium mongolicum* Regel leaf beetle *Galeruca daurica* (Coleoptera: Chrysomelidae). J. Appl. Entomol..

[42] Zhang D.W., Dai C.C., Ali A., Liu Y.Q., Pan Y., Desneux N., Lu Y.H. (2022). Lethal and sublethal effects of chlorantraniliprole on the migratory moths *Agrotis ipsilon* and *A. segetum*: New perspectives for pest management strategies. Pest Manag. Sci..

[43] Suleiman M., Ibrahim N.D., Majeed Q. (2012). Control of *Sitophilus zeamais* (Motsch) (Coleoptera: Curculionidae) on sorghum using some plant powders. Int. J. Agric. For..

[44] Kavallieratos N.G., Boukouvala M.C., Skourti A., Filintas C.S., Eleftheriadou N., Gidari D.L.S., Spinozzi E., Ferrati M., Petrelli R., Cianfaglione K. (2024). Essential oils from three Cupressaceae species as stored wheat protectants: Will they kill different developmental stages of nine noxious arthropods?. J. Stored Prod. Res..

[45] Kavallieratos N.G., Nika E.P., Skourti A., Spinozzi E., Ferrati M., Petrelli R., Maggi F., Benelli G. (2022). *Carlina acaulis* essential oil: A candidate product for agrochemical industry due to its pesticidal capacity. Ind. Crops Prod..

[46] Trematerra P., Ianiro R., Athanassiou C.G., Kavallieratos N.G. (2015). Behavioral interactions between *Sitophilus zeamais* and *Tribolium castaneum*: The first colonizer matters. J. Pest Sci..

[47] Kavallieratos N.G., Boukouvala M.C., Pappa A.P.A., Canale A., Benelli G. (2023). Being exposed to low concentrations of pirimiphos-methyl and chlorfenapyr has detrimental effects on the mobility of *Trogoderma granarium*. Pest Manag. Sci..

[48] Boukouvala M.C., Kavallieratos N.G., Maggi F., Angeloni S., Ricciutelli M., Spinozzi E., Ferrati M., Petrelli R., Canale A., Benelli G. (2023). Being exposed to *Acmella oleracea*-based insecticide extract reduces mobility and mating success in *Prostaphanus truncatus*, the major pest of maize in storages. J. Stored Prod. Res..

[49] Finney D.J. (1971). Probit Analysis.

[50] R Core Team (2017). R: A Language and Environment for Statistical Computing.

[51] Zar J.H. (2014). Biostatistical Analysis.

[52] Sall J., Lehman A., Creighton L. (2001). JMP start statistics. A Guide to Statistics and Data Analysis Using JMP and JMP in Software.

[53] Sokal R.R., Rohlf F.J. (1995). Biometry: The Principles and Practice of Statistics in Biological Research.

[54] SAS Institute Inc (2021). Using JMP 14.

[55] Kumar R. (2017). Insect Pests of Stored Grain: Biology, Behavior, and Management Strategies.

[56] Rosentrater K.A., Rosentrater K.A. (2022). Insects in grains: Identification, damage, and detection. Storage of Cereal Grains and their Products.

[57] Nguyen T.T., Collins P.J., Ebert P.R. (2015). Inheritance and characterization of strong resistance to phosphine in *Sitophilus oryzae* (L.). PLoS ONE.

[58] Manal A.A., Trandil F.W., Marwa I.M., Shawir M.S. (2017). Resistance status and associated resistance mechanisms to certain insecticides in rice weevil *Sitophilus oryzae* (Coleoptera: Curculionidae). Alex. J. Agric. Sci..

[59] de Andrade Melo Junior J.L., da Silva J.A., Santoro K.R., Badji C.A. (2018). Insecticide resistance of corn weevil populations from semi-arid regions. Aust. J. Crop Sci..

[60] Bell C.H., Motarjemi Y. (2023). Food safety assurance systems: Infestation management in food production premises. Encyclopedia of Food Safety.

[61] Boukouvala M.C., Kavallieratos N.G., Nika E.P. (2022). Insecticidal properties of etofenprox for the control of *Ephestia kuehniella*, *Rhyzopertha dominica*, *Sitophilus oryzae*, and *Tribolium confusum* on stored barley, maize, oats, rice, and wheat. Environ. Sci. Pollut. Res..

[62] He Y., Zhao J., Zheng Y., Weng Q., Biondi A., Desneux N., Wu K. (2013). Assessment of potential sublethal effects of various insecticides on key biological traits of the tobacco whitefly, *Bemisia tabaci*. Int. J. Biol. Sci..

[63] Hannig G.T., Ziegler M., Marcon P.G. (2009). Feeding cessation effects of chlorantraniliprole, a new anthranilic diamide insecticide, in comparison with several insecticides in distinct chemical classes and mode-of-action groups. Pest Manag. Sci..

[64] Baltzegar J., Jones M.S., Willcox M., Ramsey J.M., Gould F. (2023). Population genetic structure of the maize weevil, *Sitophilus zeamais*, in southern Mexico. PLoS ONE.

[65] Morrison W.R., Poling B., Leskey T.C. (2017). The consequences of sublethal exposure to insecticide on the survivorship and mobility of *Halyomorpha halys* (Hemiptera: Pentatomidae). Pest Manag. Sci..

[66] Silva Barros L., Takao Yamamoto P., Merten P., Naranjo S.E. (2020). Sublethal effects of diamide insecticides on development and flight performance of *Chloridea virescens* (Lepidoptera: Noctuidae): Implications for *Bt* soybean refuge area management. Insects.

[67] De Castro e Castro B.M., Martínez L.C., Plata-Rueda A., Soares M.A., Wilcken C.F., Zanuncio A.J.V., Fiaz M., Zanuncio J.C., Serrão J.E. (2021). Exposure to chlorantraniliprole reduces locomotion, respiration, and causes histological changes in the midgut of velvetbean caterpillar *Anticarsia gemmatalis* (Lepidoptera: Noctuidae). Chemosphere.

[68] Guedes N.M.P., Guedes R.N.C., Ferreira G.H., Silva L.B. (2009). Flight take-off and walking behavior of insecticide-susceptible and–resistant strains of *Sitophilus zeamais* exposed to deltamethrin. Bull. Entomol. Res..

[69] de Araújo A.M.N., Faroni L.R.D.A., de Oliveira J.V., do Amaral Ferraz Navarro D.M., e Silva Barbosa D.R., Breda M.O., de Franca S.M. (2017). Lethal and sublethal responses of *Sitophilus zeamais* populations to essential oils. J. Pest Sci..

[70] Athanassiou C.G., Kavallieratos N.G., Campbell J.F. (2017). Competition of three species of *Sitophilus* on rice and maize. PLoS ONE.

[71] Likhayo P.W., Hodges R.J. (2000). Field monitoring *Sitophilus zeamais* and *Sitophilus oryzae* (Coleoptera: Curculionidae) using refuge and flight traps baited with synthetic pheromone and cracked wheat. J. Stored Prod. Res..

[72] Lloyd Chesnut T. (1972). Flight habits of the maize weevil as related to field infestation of corn. J. Econ. Entomol..

[73] Giles P.H., Ashman F. (1971). A study of pre-harvest infestation of maize by *Sitophilus zeamais* Motsch.(Coleoptera: Curculionidae) in the Kenya highlands. J. Stored Prod. Res..

[74] Vásquez-Castro J.A., De Baptista G.C., Gadanha C.D., Trevizan L.R. (2012). Insecticidal effect and residual action of fenitrothion and esfenvalerate on *Sitophilus oryzae* and *S. zeamais* (Coleoptera: Curculionidae) in stored maize and wheat. Int. Sch. Res. Netw..

[75] Subramanyam B., Hagstrum D.W., Subramanyam B. (2018). Resistance measurement and management. Integrated Management of Insects in Stored products.

